# Nesfatin-1-like peptide is a novel metabolic factor that suppresses feeding, and regulates whole-body energy homeostasis in male Wistar rats

**DOI:** 10.1371/journal.pone.0178329

**Published:** 2017-05-25

**Authors:** Kavishankar Gawli, Naresh Ramesh, Suraj Unniappan

**Affiliations:** Laboratory of Integrative Neuroendocrinology, Department of Veterinary Biomedical Sciences, Western College of Veterinary Medicine, University of Saskatchewan, Saskatoon, Canada; Charité-Universitätsmedizin Berlin, Campus Benjamin Franklin, GERMANY

## Abstract

Nucleobindin-1 has high sequence similarity to nucleobindin-2, which encodes the anorectic and metabolic peptide, nesfatin-1. We previously reported a nesfatin-1-like peptide (NLP), anorectic in fish and insulinotropic in mice islet beta-like cells. The main objective of this research was to determine whether NLP is a metabolic regulator in male Wistar rats. A single intraperitoneal (IP) injection of NLP (100 μg/kg BW) decreased food intake and increased ambulatory movement, without causing any change in total activity or energy expenditure when compared to saline-treated rats. Continuous subcutaneous infusion of NLP (100 μg/kg BW) using osmotic mini-pumps for 7 days caused a reduction in food intake on days 3 and 4. Similarly, water intake was also reduced for two days (days 3 and 4) with the effect being observed during the dark phase. This was accompanied by an increased RER and energy expenditure. However, decreased whole-body fat oxidation, and total activity were observed during the long-term treatment (7 days). Body weight gain was not significantly different between control and NLP infused rats. The expression of mRNAs encoding adiponectin, resistin, ghrelin, cholecystokinin and uncoupling protein 1 (UCP1) were significantly upregulated, while leptin and peptide YY mRNA expression was downregulated in NLP-treated rats. These findings indicate that administration of NLP at 100 μg/kg BW reduces food intake and modulates whole body energy balance. In summary, NLP is a novel metabolic peptide in rats.

## Introduction

Nucleobindin 1 (NUCB1) is a widely expressed multi-domain calcium and DNA binding protein [1] that exhibits significant structural homology with a related protein, nucleobindin 2 (NUCB2). The nucleobindins share 68% conserved amino acids in humans. NUCB1 is expressed in the pituitary, thyroid, parathyroid, gastrointestinal tract, adrenals, gonads and pancreatic islets of langerhans. However, within these tissues, NUCB1 expression is not ubiquitous [2], but consistently appears to be associated with the golgi apparatus and has also been widely reported to be present within the nucleus [1, 3], endoplasmic reticulum [4, 5] and cytoplasm [6]. This distribution of NUCB1 within various endocrine tissues and cell organelles suggest a functional role for NUCB1 in multiple cellular processes. Last decade witnessed the discovery and characterization of nesfatin-1, an 82-amino acid peptide encoded in the 396-amino acid precursor, NUCB2. It was proposed that prohormone convertases (PC 1/3 or 2) processes NUCB2 into three distinct fragments: nesfatin-1 (1–82 amino acids), nesfatin-2 (85–163 aa), and nesfatin-3 (166–396 aa) [7]. Among these, nesfatin-1 alone was found to be biologically active. Nesfatin-1 (NEFA/Nucleobindin-2 encoded satiety and fat-influencing protein) is expressed in the brain, and peripheral endocrine organs, and was implicated as an appetite suppressing peptide that plays an important role in regulating feeding behavior in rats. Central or peripheral administration of full length or the mid-segment (M30) of nesfatin-1 reduces food intake and fat mass in rodents [7–9] and pigs [10]. Long-term subcutaneous infusion and IP injection of nesfatin-1 decreased food intake and modulated whole body energy homeostasis in rats [11]. However, peripheral administration of nesfatin-1 in rodents causing reduced food intake is controversial. Some other studies [12, 13] found no effects for nesfatin-1 on food intake following IP or subcutaneous injection. Despite this, nesfatin-1 is now considered a multi-functional peptide in many vertebrates, with system- and cell-specific functions, including the regulation of metabolism, cardiovascular function, stress and reproduction [9, 11, 14–18]. Does NUCB1 (closely related to the nesfatin-1 precursor, NUCB2) also encode a nesfatin-1-like molecule? Our efforts in the past year made significant progress in addressing the above question, and we reported a nesfatin-1-like peptide (NLP) in mice [19], zebrafish and goldfish [20].

*In silico* analysis of NUCB1 amino acid sequence shows that it contains an N-terminal signal peptide sequence, followed by a “nesfatin-1-like peptide” region flanked by signature sequences representing proprotein convertase cleavage sites, a feature that it shares with NUCB2. *In silico* analysis identified that nesfatin-1-like peptide could be processed from NUCB1, producing NLP in mammals (19), zebrafish and goldfish (20). NUCB1 is also a secreted peptide due to the presence of a signal peptide (19, 20). The NLP sequence shows a greater degree of similarity, and is more highly conserved across rat, mouse, human, fish and frog within the region corresponding to the proposed bioactive core of nesfatin-1 [19]. Immunofluorescence studies revealed the presence of NLP in the endocrine pancreas of mice [19], brain, pituitary, gut, ovary and testis of fish [20]. NLP was reported to possess satiety effects with tissue and cell specific expression in response to metabolic and reproductive regulators, suggesting a role in metabolism and reproduction [20]. NUCB1 is a regulator of amyloid fibril formation [21, 22], autoimmunity [23], apoptosis [24], calcium homeostasis [25], and bone matrix maturation [26]. NLP is insulinotropic in mice (19) and anorectic in fish (20). Overall, NUCB1/NLP has multiple biological functions in vertebrates.

Like nesfatin-1, is NLP a metabolic peptide? Does it alter metabolic hormones? We carried out an in-depth analysis of the metabolic effects of both acute and chronic administration of NLP on lean rats. It was found that NLP indeed has anorectic effects, and it modulates whole body energy homeostasis, when administered intraperitoneally, or *via* subcutaneous infusion. We also found that NLP alters the expression of mRNAs encoding key hormones involved in the regulation of food intake and energy balance. This research provides new information, and introduces NLP as a novel regulator of whole body energy homeostasis.

## Materials and methods

### Animals

Male Wistar rats were purchased from Charles River Laboratories Inc. (Saint-Constant, Quebec, Canada). Rats were individually housed in polycarbonate cages with bedding in a 12 h light (0700–1900 h):12 h dark (1900–0700) photoperiod under controlled temperature (23 ± 1°C) and humidity. Animals had *ad libitum* access to normal rat chow (Purina Mills, St. Louis, Missouri) and tap water, and were randomized by body weight, unless stated otherwise. All procedures and protocols used in *in vivo* studies adhered to the guidelines of the Canadian Council for Animal Care, and were approved by the University of Saskatchewan Animal Care Committee (Protocol Number 2012–0033).

### NLP Administration

#### Materials

Rat NLP (VPVDRAAPHQEDNQATETPDTGLYYHRYLQEVINVLETDGHFREKLQ AANAEDIKSGKLSQELDFVSHNVRTKLDEL), with no post-translational modifications, was synthesized and purified (> 95% purity) by Abgent Technologies (California, USA). NLP was freshly reconstituted in 0.9% sterile saline (0.9% sodium chloride) for each study. Seven day (Model 2ML1) Alzet osmotic mini-pumps were purchased from Durect Corporation (Cupertino, California).

#### Acute effects of NLP on metabolism—Intraperitoneal injection

Rats (average body weight = 205±3 g) were housed and acclimated as reported earlier [9, 11, 27, 28]. Body weight of rats were recorded before the IP injection and implantation of osmotic mini-pumps. Rats (n = 6/group) were IP injected once, just before the commencement of dark phase (between 1800 to 1900 h) either with 200 μL of sterile saline (control group) or 100 μg/kg BW/day of NLP (treated group), using a 1 mL syringe attached to a 27-guage needle (Becton-Dickinson, Ontario, Canada). Our objective was to determine whether NLP modulates feeding and metabolism at a dose identical to the concentration of nesfatin-1 that was found metabolically active in rats. Therefore, we chose 100 μg/kg BW/day, a dose previously validated for metabolic functions in rats [9, 11]. Rats were transferred to the Comprehensive Laboratory Animal Monitoring System (CLAMS; Columbus Instruments, Ohio) cages to acclimate, and monitor feeding and metabolic parameters for 4 days before the first study. The CLAMS gas sensors and balances were calibrated as per manufacturer’s guidelines,prior to monitoring the metabolic parameters. Individual cages in the CLAMS system were connected to an open-circuit calorimeter for determination of oxygen consumption (VO_2_), CO_2_ production (VCO_2_), respiratory exchange ratio (RER), heat, food, and water intake, and activity (horizontal, vertical and ambulatory movements). Energy expenditure (EE) was determined by multiplying calorific value with VO_2_ (EE = CV * VO_2_) and CV by using the following equation: CV = (3.815 + 1.232) * RER. The energy derived from carbohydrates and fats were extrapolated from RER data by employing the methods of McLean and Tobin, using the equations and methods described in the protocols provided by Columbus instruments (http://www.colinst.com). The experiments were repeated using second cohort of rat’s weight matched with the first cohort rats, with similar results obtained. The data provided here are combined from two different experiments (total n = 6 rats/group).

#### Chronic effects of NLP on metabolism—Subcutaneous infusion using osmotic mini-pumps

The rats were allowed to remain in their cages for next 3 days in order to wash out the injected peptide. After this second acclimation period of 3 days, the same rats (average body weight = 250±7 g) were used for implanting the 7-day osmotic mini-pump containing NLP. To test the long-term effects of NLP on food intake and other metabolic variables, the pumps were carefully loaded with 0.9% saline (vehicle), or NLP (100 μg/kg/BW/day) and were incubated at 37°C for at least 3 h to achieve the osmotic functioning of the pump. The subcutaneous implantation of the osmotic pumps was performed as described previously [11]. Using CLAMS, food intake and other metabolic parameters were obtained continuously over 24 h (0600 to 0600 h next day) daily during the 7-day experimental period. Food and water intake data were analyzed for individual days (dark and light phases), and, metabolic parameters: RER, heat, EE, energy derived from CHO and fat, VO_2_ and VCO_2_ were measured for all 7 days.

### Tissue collection and processing

To determine whether exogenous administration of NLP has any influence on the expression of metabolic hormone encoding mRNAs; stomach, small, and large intestine, pancreas and adipose (white and brown) tissue were collected. Post-experiment (long-term study), animals were euthanized by isoflurane anesthesia. Tissue collection started at noon, and was finished within 2 hours. Blood was drawn from the heart and the tissues of interest were excised, and flash frozen in liquid nitrogen and stored at -80°C. Total RNA was extracted from 10 mg tissue homogenate using TRIzol reagent (Invitrogen Canada Inc., Ontario, Canada) or Aurum total RNA kit (Bio-Rad Laboratories Inc., Ontario, Canada) for fat tissue, as per the instructions of the manufacturer. Concentration and purity of RNA was determined using NanoDrop 2000c (Thermo, Vantaa, Finland) and complementary DNA was synthesized using iScript cDNA synthesis kit (Bio-Rad laboratories, Inc., Ontario, Canada) and quantified by real-time RT-qPCR.

### Gene expression analysis

The primer sequences and PCR conditions used for quantifying mRNA expression are listed in Table 1. Gene expression was measured using RT- quantitative PCR with SYBR Green Master Mix, and the CFX Connect Real-Time PCR Detection System (Bio-Rad) and normalized to the expression of beta-actin as a housekeeping gene, using the Livak method [29]. Before sample analysis, high efficiency and annealing temperatures of the primers were achieved by validation and optimization. Samples were run in duplicate with the absence of template DNA in the reaction mixture being considered negative control. A melt curve analysis was performed after the amplification phase at 65°C to 95°C with a 0.5°C increment, enabling amplification of a single product devoid of dimer formation or artifacts by each primer pair set.

**Table 1:** Sequences of forward and reverse primers, and the conditions employed in PCR and RT-qPCR analyses of the expression of mRNAs of interest.

Gene	Sequence (5’ to 3’)	Annealing [Temp (sec)]
**β-actin**	Sense -TGACAGGATGCAGAAGGAGATT	61.0°C (30 s)
	Anti-sense—AAACGCAGCTCAGTAACAGTC	
**Adiponectin**	Sense—TTCACCTACGACCAGTATCAGG	60.0°C (30 s)
	Anti-sense—TACGGGCTGCTCTGAATTAGT	
**Resistin**	Sense—AGAAGGCACAACCGTCACTA	60.0°C (30 s)
	Anti-sense—GGGCAAGCTCAGTTCTCAAT	
**Leptin**	Sense—GGCATTCCTTCTGTTTCTAGGT	60.0°C (30 s)
	Anti-sense—TGGTCTTGATGAGGGTTTTGGT	
**Ghrelin**	Sense—GAAAGCCCAGCAGAGAAAGGAA	61.0°C (30 s)
	Anti-sense—CCAACATCGAAGGGAGCATTGA	
**Cholecystokinin**	Sense—CTGAGGACTACGAATACCCATC	64.5°C (30 s)
	Anti-sense—AGCATAGCAACATTAGGTCTGG	
**Polypeptide YY**	Sense—AGCGGTATGGGAAAAGAGAAGT	61.0°C (30 s)
	Anti-sense—GCAAGTGAAGTCGGTGTAGTT	
**UCP1**	Sense—TTAAAGAGCGAGAGGAAGGGAC	63.3°C (30 s)
	Anti-sense—GGGAAGGTGATGATGTCTGCTA	
**Insulin**	Sense—AGCGTGGATTCTTCTACACAC	64.5°C (30 s)
	Anti-sense—TTATTCATTGCAGAGGGGTGGA	
**Glucagon**	Sense—CATCGTGGCTGGATTGTTTGT	63.3°C (30 s)
	Anti-sense—TTGTTCCGGTTCCTCTTGGT	

**PCR condition:** 95°C (30 s), 35 cycles of 95°C (10 s); Annealing Temp (secs).

### Statistical analyses

Statistical analyses were performed with GraphPad Prism (GraphPad Software, Inc.). Analyses of data pertaining to CLAMS study, RT-qPCR and group comparison were conducted using One-way ANOVA followed by Student’s ‘*t’* test. Two-way ANOVA followed by Bonferroni post-hoc test was used for testing groups with different time points. *P < 0.05; **P < 0.01; ***P<0.001 were considered statistically significant. Data are expressed as mean ± SEM.

## Results

### Intraperitoneal injection of NLP influences metabolic parameters

**Fig 1:**
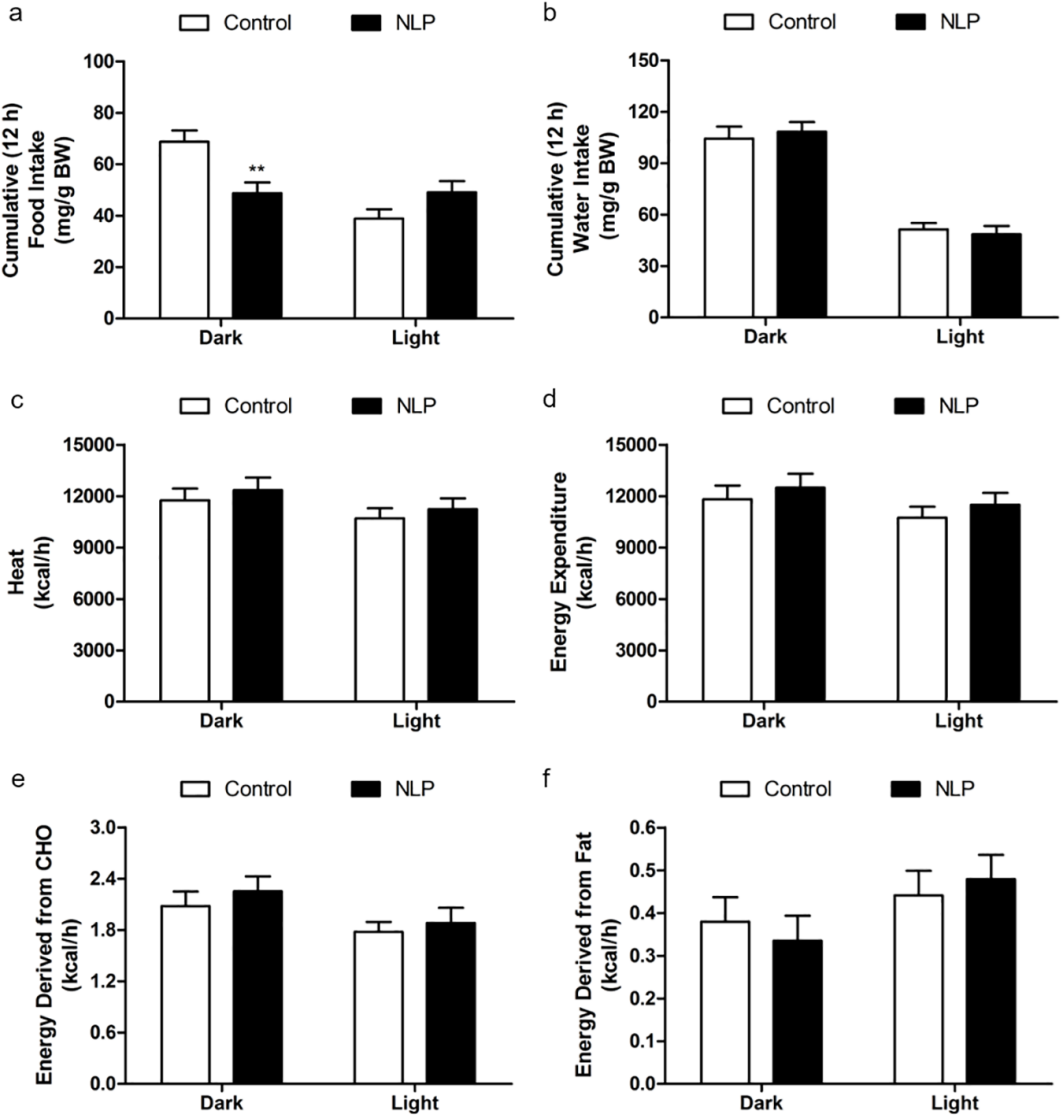
Intraperitoneal injection of NLP caused anorectic effect with no change in energy expenditure. NLP was injected at 100 μg/kg BW before the dark phase. A decrease in food intake, but no significant difference in water intake (mg/g BW; a & b) was observed. No significant changes in heat (c), EE (d) or relative contribution of carbohydrates (e) or fat (f) towards energy expenditure was observed during both dark and light phase, expressed as kcal/h. Data are represented as mean ± SEM with n = 6 rats/group. **P < 0.01, compared to control.

**Fig 2:**
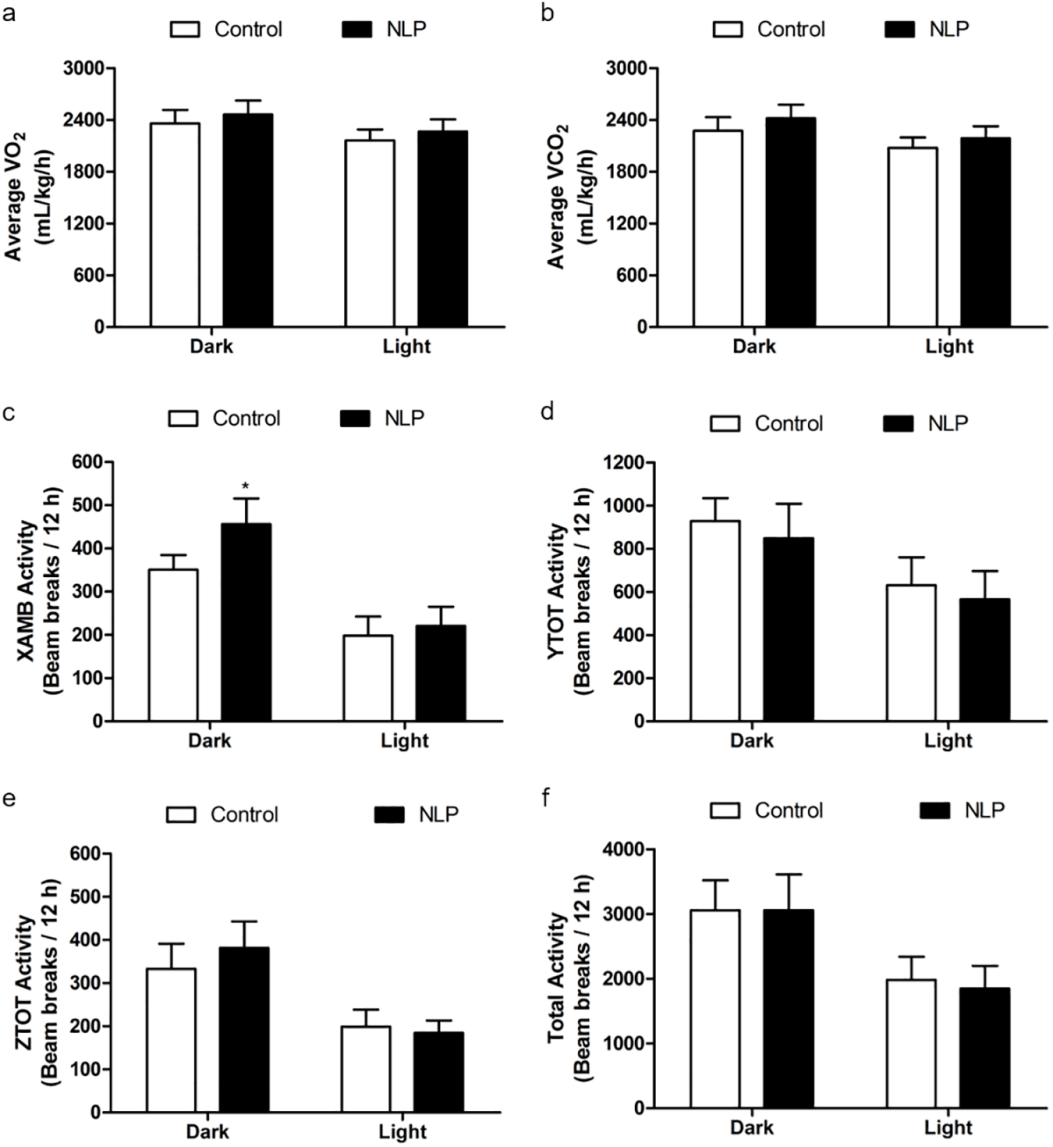
Intraperitoneal injection of NLP had no effect on physical activity during both dark and light phase. No significant change in average O_2_ consumption and CO_2_ production was observed during the dark and light phase (a & b). The locomotor activity (c-f); Ambulatory (X-AMB, refers to beam breaks in X axis) was higher in NLP treated group, but no change in horizontal (Y-TOT), vertical (Z-TOT) movements or in total activity was observed after the treatment. The activity is presented as beam breaks/12 h. Data are represented as mean ± SEM with n = 6 rats/group.

NLP injected IP before the dark phase caused a significant reduction in food intake when compared to saline treated group during the 12 h dark phase and the effect did not last till the next light phase **(Fig 1A)**. There was no significant change in water intake **(Fig 1B)** or heat **(Fig 1C)** of NLP treated group in the dark or light phase. No significant change in the total energy expenditure was observed in NLP treated animals when compared to saline controls **(Fig 1D)**. The relative contribution of carbohydrate (CHO) towards total energy production was similar in NLP treated animals compared to controls **(Fig 1E)**. Fatty acid oxidation was also found to be unchanged after NLP injection compared to saline injected controls **(Fig 1F)**. There were no noticeable changes in oxygen consumption (VO_2_), and CO_2_ production (VCO_2_) in NLP treated rats **(Fig 2A and 2B)**. Ambulatory **(Fig 2C)** movement was significantly higher after NLP administration, whereas, no change in the vertical **(Fig 2D)** or horizontal activities **(Fig 2E)** was observed when compared to controls. However, the total activity was not different between the control and treated groups during both dark and light phase **(Fig 2F)**.

### Subcutaneous NLP infusion for 7 days modulates feeding and metabolism

**Fig 3:**
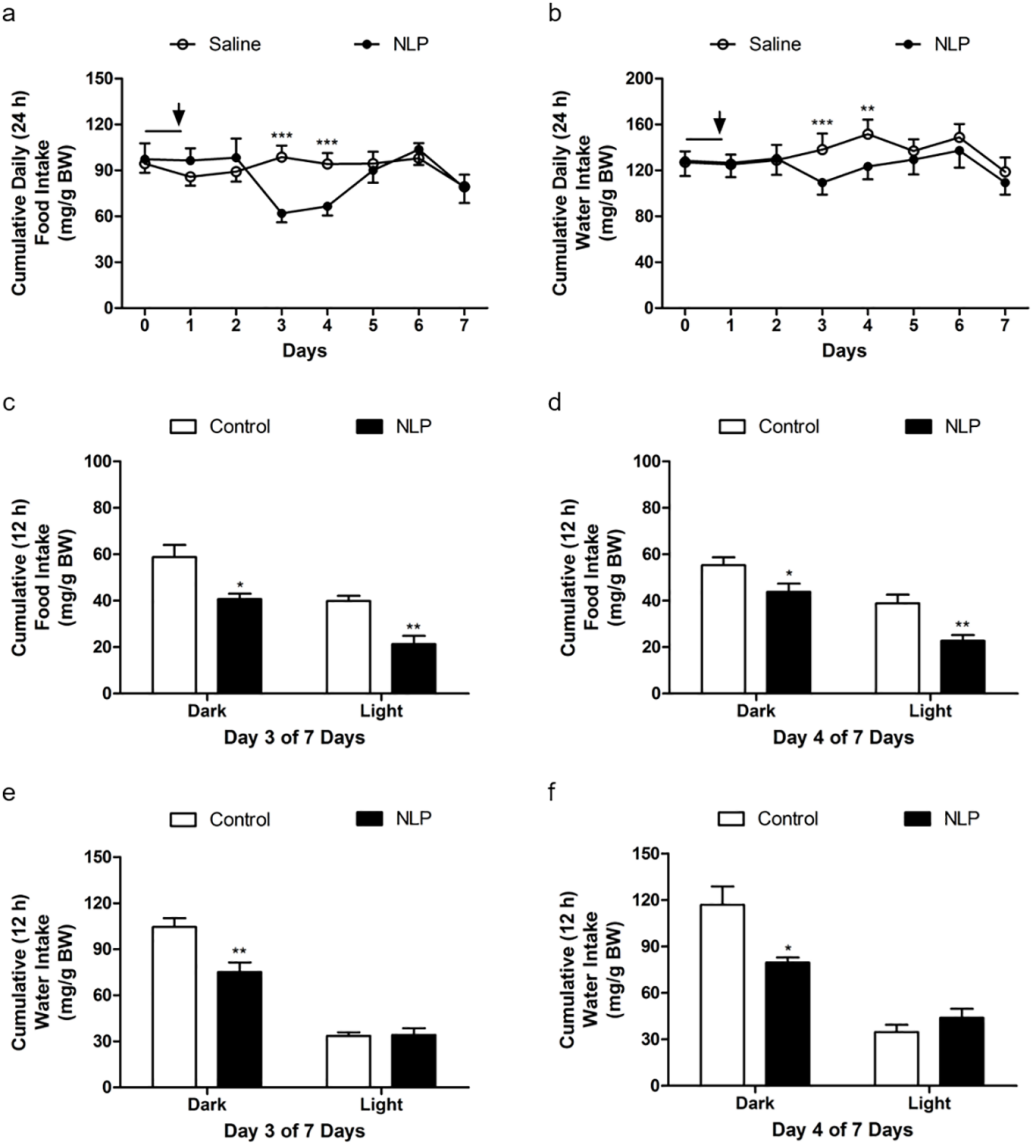
**Continuous peripheral administration of NLP at 100** μ**g/kg BW/h/day for 7 days reduced food and water intake (mg/g BW; a & b).** The black line indicates the last day of wash out period after the IP injection study, and the downward arrow indicates the pump implantation. The anorexigenic nature of NLP was found in days 3 and 4 of 7 days. A significant difference in food intake between NLP and saline treated group was observed in both dark and light phase. Water intake was significantly reduced in NLP treated group during dark phase on day 3 and day 4. All data are represented as means ± SEM with n = 6 rats/group. *P < 0.05; **P < 0.01; ***P<0.001 compared to control.

**Fig 4:**
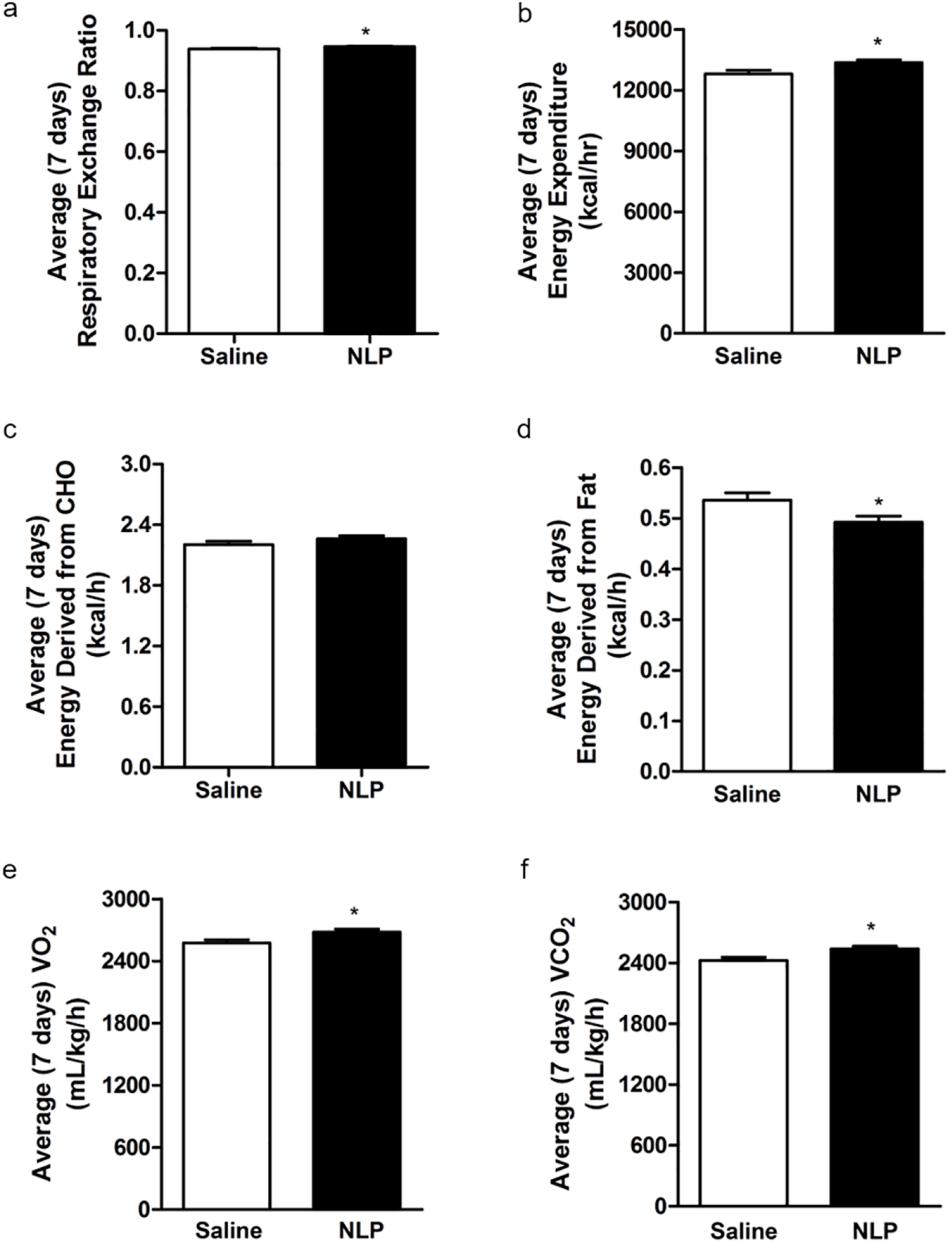
**Respiratory exchange ratio (RER; a) and total energy expenditure (EE; b) were increased during the 7-day continuous infusion of NLP.** The relative contribution of carbohydrate (CHO; c) had no change, but a decrease in fatty acid oxidation (d) was observed during the treatment. With an increase in EE, average O_2_ consumption (e) and CO_2_ production (f) were increased in NLP treated rats. All data are represented as means ± SEM with n = 6 rats/group. *P < 0.05 compared to control.

**Fig 5:**
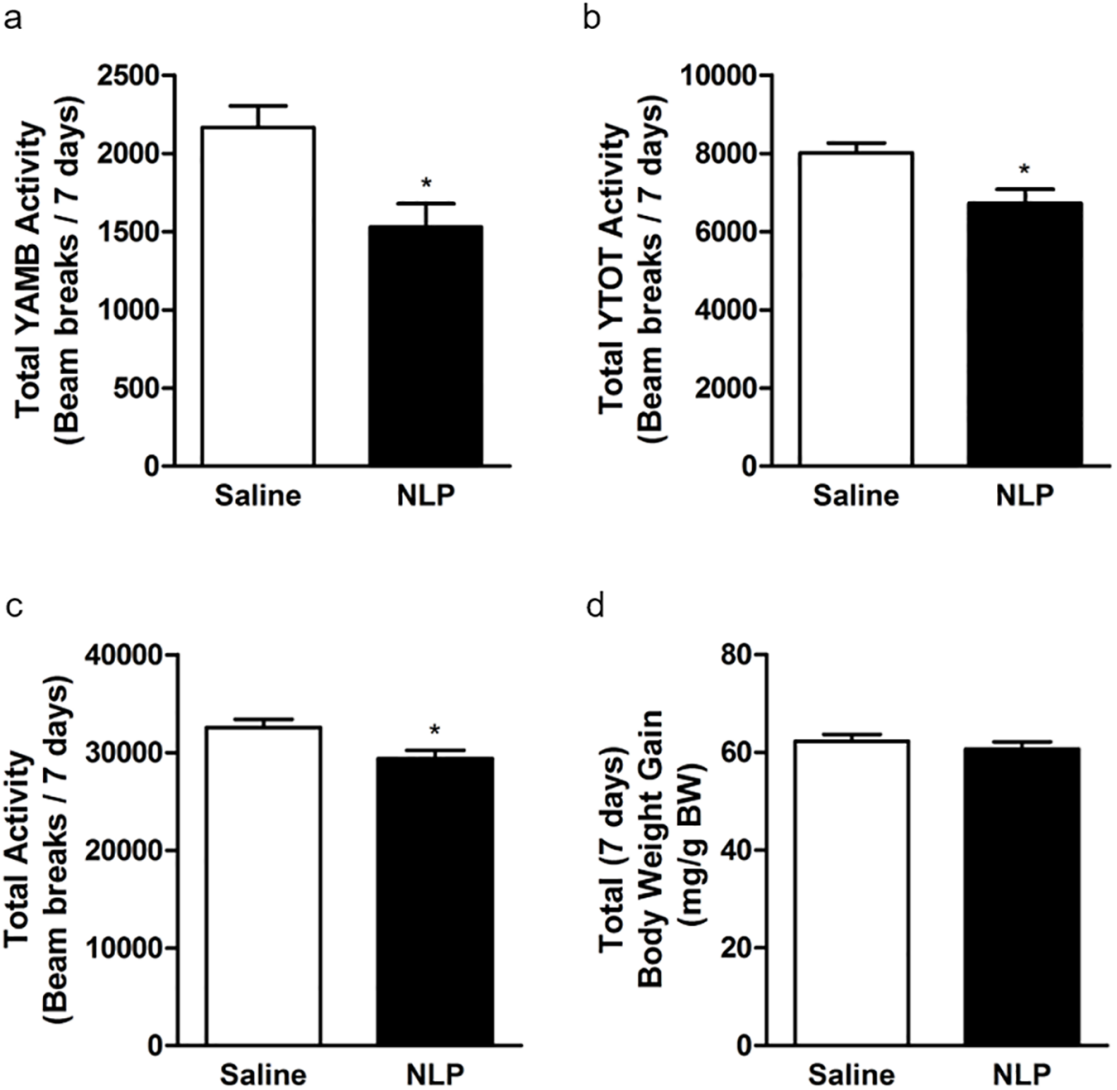
**The locomotor activity (a-c); ambulatory (Y-AMB, refers to successive beam breaks in Y axis) and horizontal (Y-TOT) movements were decreased.** Total activity (X+Y+Z+AMB) was reduced in NLP treated animals (beam breaks/7 days). No change in total body weight (mg/g BW; d) was observed after the 7-day treatment. Data are represented as mean ± SEM with n = 6 rats/group. *P < 0.05, compared to control.

NLP treated rats showed a significant reduction in food intake for two days (day 3 and 4) compared to saline treated rats (Fig 3A). Similar results were found with water intake (Fig 3B), where NLP treated rats consumed less water compared to controls. We found that the anorectic effect of NLP lasted only for two days. However, a significant difference in food intake between NLP- and saline-treated group was observed during both dark and light phase (Fig 3C). The same anorectic effect was observed on the following day (Fig 3D). On the other hand, water intake was significantly reduced in NLP-treated group on day 3 (Fig 3E) and day 4 (Fig 3F). No change in heat was found (S1A Fig), but a significant increase in RER of NLP treated rats was observed (Fig 4A). The total energy expenditure of NLP infused rats was higher compared to controls (Fig 4B), and the relative contribution of CHO towards energy production was found to be same in both groups (Fig 4C). However, the energy derived from fat was significantly less in NLP-treated rats compared to the controls (Fig 4D). Oxygen consumption and CO_2_ production was significantly higher in NLP infused rats when compared to saline infused rats (Fig 4E and 4F). No change in cumulative oxygen consumption and CO_2_ production was observed, which is consistent with the results found after IP injection (S1B and S1C Fig). Also, no changes in locomotor activity; horizontal (XTOT), vertical (ZTOT) and ambulatory (X-AMB) were observed between saline and NLP treated rats (S1D–S1F Fig). Ambulatory (Fig 5A), YTOT activity (Fig 5B) and total activity (Fig 5C) remained significantly lower in NLP treated animals. The body weight gain (Fig 5D) of rats during the 7-day period in both groups was not significantly different.

### NLP influences mRNAs encoding metabolic hormones

**Fig 6:**
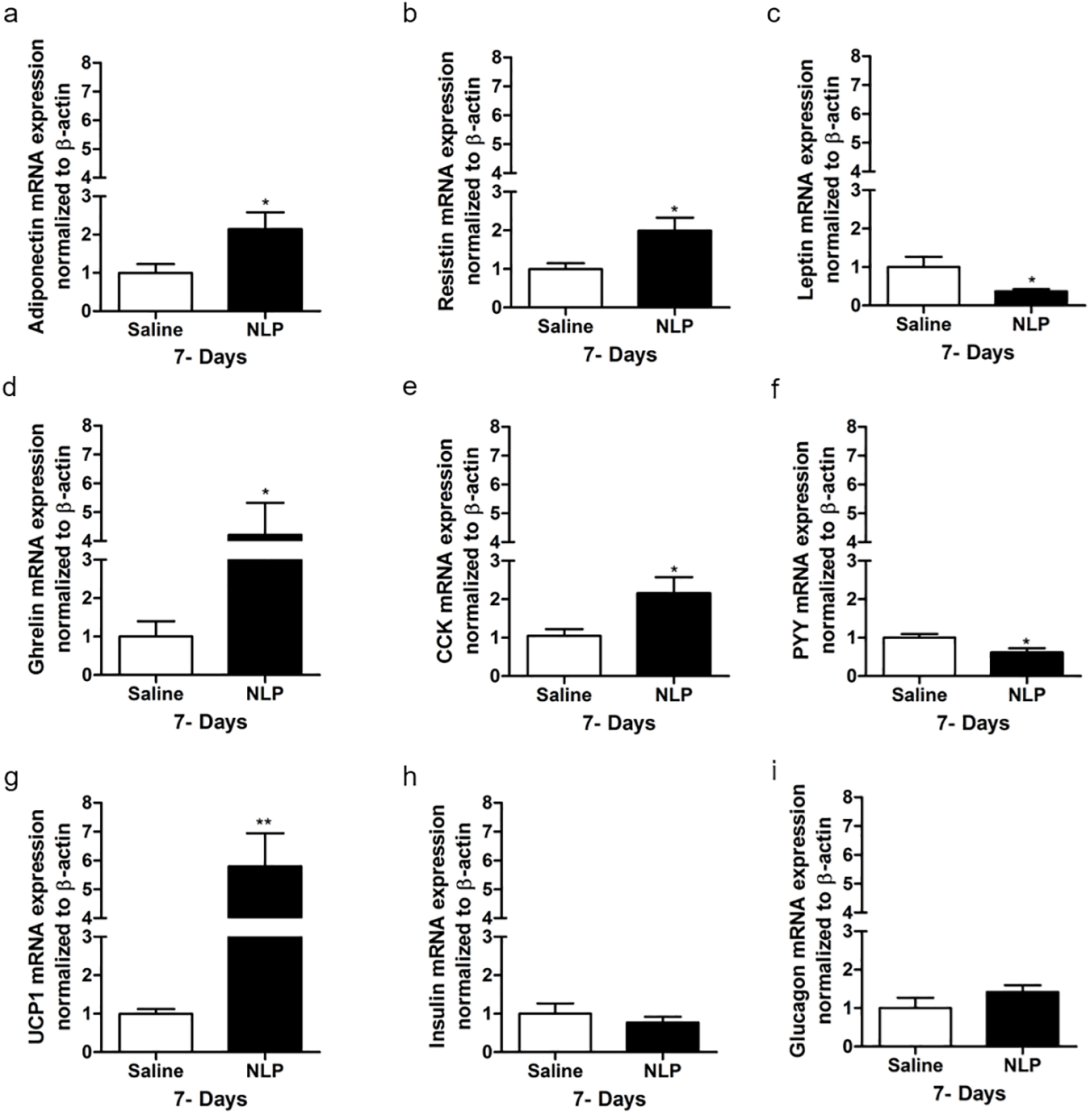
Intraperitoneal and peripheral administration of NLP at 100 μg/kg BW significantly regulated metabolic hormones in different tissues. NLP treatment upregulated adiponectin (a) and resistin (b), while downregulated leptin (c) mRNA expression in white adipose tissue (WAT). The mRNA expression of ghrelin (d) and CCK (e) was increased in stomach and small intestine (duodenum) respectively, whereas, PYY (f) expression in large intestine was found decreased. Increase in UCP1 (g) and no change in the mRNA expression of insulin (h) and glucagon (i) was observed in NLP treated animals. All data are represented as mean ± SEM with n = 6 rats/group. Samples were analyzed in duplicates. *P < 0.05, **P < 0.01 compared to saline treated rat.

Compared to the saline treated rats, the expression of adiponectin and resistin (Fig 6A and 6B) was significantly increased in white adipose tissue (WAT), but the mRNA expression of leptin (Fig 6C) was reduced, in NLP treated animals. In addition to this, ghrelin mRNA expression in the stomach (Fig 6D), and CCK mRNA expression (Fig 6E) in small intestine, was significantly upregulated. PYY mRNA expression in the large intestine (Fig 6F) was downregulated in NLP treated animals. UCP1 mRNA expression in the brown adipose tissue (BAT) was 6-fold higher than the controls (Fig 6G). No changes in the insulin and glucagon mRNA expression in the pancreas were observed (Fig 6H and 6I) in rats treated with NLP.

## Discussion

Nesfatin-1, present in *NUCB2*, is an anorectic peptide in rats [7]. We discovered anorectic effects for the *NUCB1* encoded NLP in fish [20]. The results presented here extend these findings, and indicate that NLP possess similar anorectic effects in rats. In the acute study, we found NLP eliciting a reduction in food intake. The absence of any differences in feeding in the light phase is likely due to compensatory eating in response to reduced eating in the dark phase. Further studies are required to determine whether NLP influences light phase specific food intake and other aspects of energy balance if administered prior to the onset of light cycle. The degree of anorectic effects of NLP found in this study is comparable to that of nesfatin-1 [9, 11, 30]. IP injection also resulted in an increase in ambulatory activity, an aspect that was influenced by nesfatin-1 as well. Together, these results indicate that NLP resembles nesfatin-1 in its effects on food intake.

Chronic administration of NLP resulted in decreased food intake for two days (day 3 and 4) during the 7-day period. The normalization of feeding found after day 4 is likely due to compensatory responses and/or receptor desensitization or downregulation during continuous infusion. Additional studies are required to unravel the short-lived anorectic effects found here. During the 7-day infusion, we found an increase in RER. Previous studies [9, 11] reported that nesfatin-1 treated rats had decreased RER, and used fat as a primary source of energy. While in this study, we observed decreased fat oxidation after chronic administration of NLP without causing any change in carbohydrate utilization. Total activity was decreased despite an increase in energy expenditure during subcutaneous infusion period only. This effect was not observed following NLP IP injection, and one possible reason for this is the fast clearance of NLP after an injection. There appears to be differences in NLP actions compared to nesfatin-1. For example, one study [11] reported an increase in total activity without any change in energy expenditure on days 6–7 during the 7-day experimental period after peripheral infusion in Fischer 344 rats. Meanwhile, in another study, intracerebroventricular administration of nesfatin-1 was shown to cause an increase in EE [30] and no change in physical activity in male Wistar rats [31]. The variations in effects of nesfatin-1, and actions of NLP in comparison to nesfatin-1 are likely due to differences in species used, the route of administration (central vs. peripheral) of the peptide and duration (short/long-term) of the study. It is possible that multiple receptors mediate the metabolic effects of nesfatin-1 and NLP.

Food intake and energy homeostasis are regulated by the brain in response to signals released from the gut, pancreas and adipose tissue [32, 33]. These signals influence local sensory nerves travelling from the periphery to the hindbrain to produce a negative or positive effect on energy balance [32]. It is likely that, NLP conveys its anorectic effect by modulating hormones involved in the regulation of food intake and energy balance. We studied the possible influence of NLP on other metabolic hormones in our next set of experiments. Following the long-term infusion of NLP, expression of preproghrelin mRNA in the stomach was found to be increased. NLP induced reduction in food intake is a reason why proproghrelin mRNA, which encodes the orexigen ghrelin is upregulated. A two-fold increase in CCK mRNA expression in the upper small intestine (duodenum) was observed in NLP treated rats. CCK is an anorexigen [34]. PYY mRNA expression was downregulated in the large intestine of NLP treated rats. PYY exists in two forms, of which one (PYY_1-36_) is orexigenic, while the other form (PYY_3-36_) is anorectic. Additional studies are required to identify how NLP changes these two forms of PYY in circulation, and its relative abundance in rats. These findings are in agreement with the peripheral effects of nesfatin-1, that is shown to upregulate CCK, while downregulate PYY in mice [35]. Our data suggest that NLP mediates these short-term appetite regulatory signals to maintain body weight and energy balance.

We explored whether NLP influences long-term metabolic signals arising from the pancreas and adipose tissue, and its impact on body weight. There were no changes in the expression of pancreatic insulin and glucagon mRNA expression after 7 days of NLP treatment. Acute peripheral administration of nesfatin-1 exerts an insulinotropic action in mice and MIN6 cells [36]. Similarly, NLP has insulinotropic effects in mice and MIN6 cells [19]. Further studies are required to elucidate the *in vivo* effects of NLP on insulin secretion and glucose homeostasis. We found reduced expression of leptin mRNA, and an increased adiponectin and resistin mRNA expression in the WAT of NLP treated rats. Leptin influences loss of fat mass and body weight [37], whereas, adiponectin administration causes a reduction in body weight [38–40]. Tissue specific protein expression and circulating levels of these peptides are necessary before making solid conclusions. However, it is very evident that NLP influences the endocrine milieu regulating long-term energy balance. A significant increase in UCP1 mRNA expression in brown fat was observed. This upregulation of UCP1 in brown fat could be a contributing factor for increased EE and thermogenesis in NLP treated rats. Also, loss of brown fat by a UCP1 promoter-driven toxin causes an increased propensity to weight gain [41] and increased fat accumulation [42] in experimental animals. Thus, UCP1 is another candidate molecule mediating NLP actions on energy balance.

## Conclusions

Our findings provide evidence for NLP as a novel anorexigenic peptide that modulates various metabolic parameters in rats. When administered peripherally, NLP reduces feeding, and modulates energy expenditure and physical activity. At least at the mRNA level, NLP regulates key metabolic hormones. The metabolic actions and effects of NLP on hormonal regulators of metabolism are comparable to that of the effects of nesfatin-1. However, some differences in such effects of NLP were also found. Both nesfatin-1 and NLP are orphan ligands, and NLP is a novel bioactive molecule. Future studies on identification of endogenous NLP, NUCB1 processing mechanisms that result in NLP, mechanism of NLP action, dose, time, tissue and species-dependent effects, and the pathways that mediate NLP induced satiety and alterations in metabolism warrant consideration.

## Supporting information

S1 Fig No significant change in heat (a) was observed. Cumulative O_2_ consumption (b) and CO_2_ production (c) remained same in both the groups. Also, no change in the locomotor activity (d-f); horizontal (XTOT), vertical (ZTOT) and ambulatory (X-AMB, refers to beam breaks in X axis) was observed between saline and NLP treated rats after 7-day study. Data are represented as mean ± SEM with n = 6 rats/group.
(TIF)
