# Supplementary figures and images for: Nesfatin-1-like peptide is a novel metabolic factor that suppresses feeding, and regulates whole-body energy homeostasis in male Wistar rats
Source: PLoS One. 2017 May 25;12(5):e0178329. doi: 10.1371/journal.pone.0178329 (PMC5444818; doi:10.1371/journal.pone.0178329)

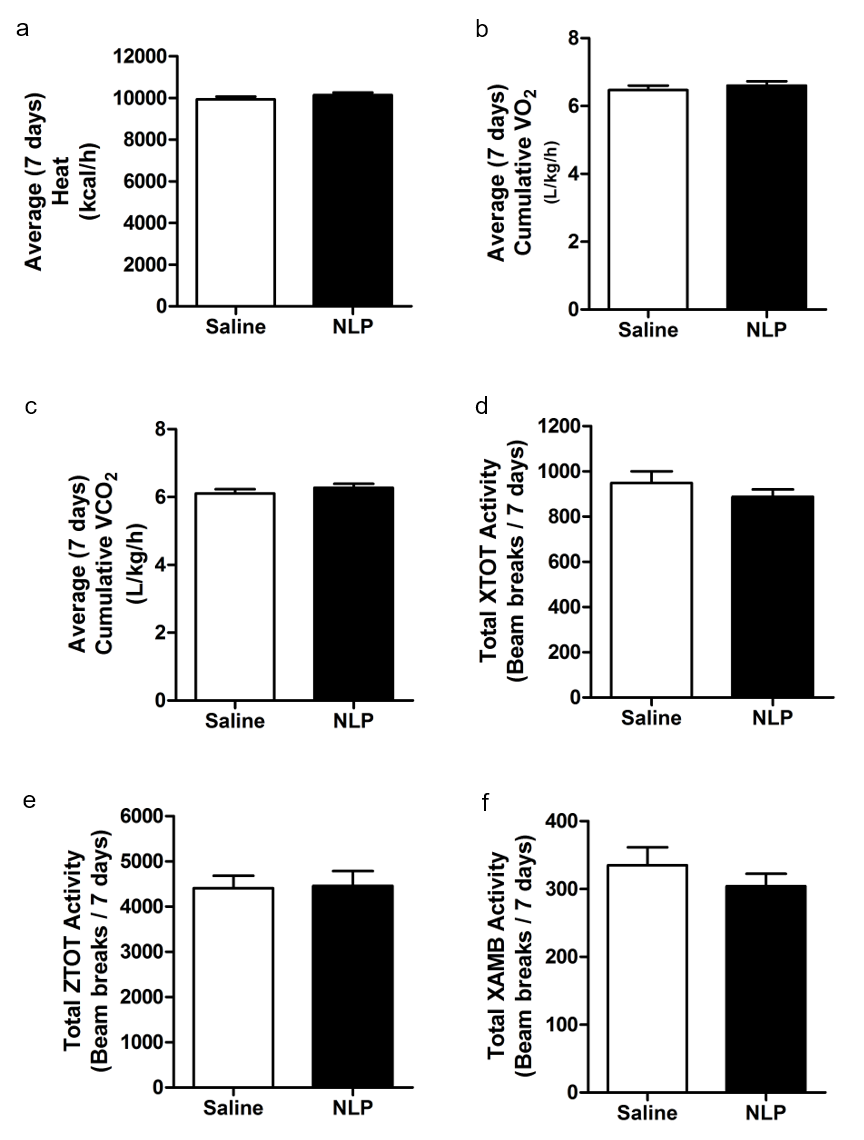

Supplement: S1 Fig — No significant change in heat (a) was observed. Cumulative O2 consumption (b) and CO2 production (c) remained same in both the groups. Also, no change in the locomotor activity (d-f); horizontal (XTOT), vertical (ZTOT) and ambulatory (X-AMB, refers to beam breaks in X axis) was observed between saline and NLP treated rats after 7-day study. Data are represented as mean ± SEM with n = 6 rats/group. (TIF) [file pone.0178329.s001.tif]
